# Effects of hyperventilation length on muscle sympathetic nerve activity in healthy humans simulating periodic breathing

**DOI:** 10.3389/fphys.2022.934372

**Published:** 2022-09-05

**Authors:** Jens Spiesshoefer, Alberto Giannoni, Chiara Borrelli, Paolo Sciarrone, Imke Husstedt, Michele Emdin, Claudio Passino, Florian Kahles, Tye Dawood, Binaya Regmi, Matthew Naughton, Michael Dreher, Matthias Boentert, Vaughan G. Macefield

**Affiliations:** ^1^ Institute of Life Sciences, Scuola Superiore Sant’Anna, Pisa, Italy; ^2^ Department of Pneumology and Intensive Care Medicine, University Hospital RWTH Aachen, Aachen, Germany; ^3^ Fondazione Toscana Gabriele Monasterio, Pisa, Italy; ^4^ Department of Neurology with Institute for Translational Neurology, University of Muenster, Muenster, Germany; ^5^ Department of Cardiology and Vascular Medicine, University Hospital RWTH Aachen, Aachen, Germany; ^6^ Human Autonomic Neurophysiology Laboratory, Baker Heart and Diabetes Institute, Melbourne, VIC, Australia; ^7^ Department of Respiratory Medicine, The Alfred Hospital, Melbourne, VIC, Australia; ^8^ Department of Medicine, Monash University, Melbourne, VIC, Australia; ^9^ Department of Medicine, UKM Marienhospital Steinfurt, Steinfurt, Germany; ^10^ Department of Anatomy & Physiology, University of Melbourne, Melbourne, VIC, Australia

**Keywords:** central sleep apnea, physiology, muscle sympathetic nerve activity, risk factor, hyperventilation

## Abstract

**Background:** Periodic breathing (PB) is a cyclical breathing pattern composed of alternating periods of hyperventilation (hyperpnea, HP) and central apnea (CA). Differences in PB phenotypes mainly reside in HP length. Given that respiration modulates muscle sympathetic nerve activity (MSNA), which decreases during HP and increases during CA, the net effects of PB on MSNA may critically depend on HP length.

**Objectives:** We hypothesized that PB with shorter periods of HP is associated with increased MSNA and decreased heart rate variability.

**Methods:** 10 healthy participants underwent microelectrode recordings of MSNA from the common peroneal nerve along with non-invasive recording of HRV, blood pressure and respiration. Following a 10-min period of tidal breathing, participants were asked to simulate PB for 3 min following a computed respiratory waveform that emulated two PB patterns, comprising a constant CA of 20 s duration and HP of two different lengths: short (20 s) vs long (40 s). Results: Compared to (3 min of) normal breathing, simulated PB with short HP resulted in a marked increase in mean and maximum MSNA amplitude (from 3.2 ± 0.8 to 3.4 ± 0.8 µV, *p* = 0.04; from 3.8 ± 0.9 to 4.3 ± 1.1 µV, *p* = 0.04, respectively). This was paralleled by an increase in LF/HF ratio of heart rate variability (from 0.9 ± 0.5 to 2.0 ± 1.3; *p* = 0.04). In contrast, MSNA response to simulated PB with long HP did not change as compared to normal breathing. Single CA events consistently resulted in markedly increased MSNA (all *p* < 0.01) when compared to the preceding HPs, while periods of HP, regardless of duration, decreased MSNA (*p* < 0.05) when compared to normal breathing.

**Conclusion:** Overall, the net effects of PB in healthy subjects over time on MSNA are dependent on the relative duration of HP: increased sympathetic outflow is seen during PB with a short but not with a long period of HP.

## 1 Introduction

Periodic breathing (PB) is a cyclical pattern of ventilation characterised by alternating phases of hyperventilation (hyperpnea, HP) and transient cessation of respiratory effort and breathing, i. e. central apnea (CA) (Somers et al., 1989a; Hall et al., 1996; Solin et al., 2003; Giannoni et al., 2016; Giannoni et al., 2019a). Beyond occurring at high altitude, PB is frequently found in patients with systolic heart failure (Heart failure with reduced ejection fraction; HFrEF), where phases of hyperventilation alternate with CA in a crescendo-decrescendo pattern that is known as Hunter-Cheyne-Stokes respiration (Somers et al., 1989a; Hall et al., 1996; Solin et al., 2003; Giannoni et al., 2016; Giannoni et al., 2019a).

Apneic events are known to increase sympathetic outflow as measured by intraneural recording of muscle sympathetic nerve activity (MSNA), reflecting chemoreflex stimulation by hypercapnia and hypoxia and (possibly) loss of ventilation-related vagal inputs (Solin et al., 2003; Giannoni et al., 2016; Giannoni et al., 2019a). Chemoreflex hypersensitivity is often observed and usually considered the main mechanism behind Cheyne-Stokes respiration (Somers et al., 1989a; Somers et al., 1989b; Hall et al., 1996; Wedewardt et al., 2010; Seitz et al., 2013) On the contrary, HP is usually accompanied by a decrease in sympathetic outflow, at least in healthy individuals (Oldenburg et al., 2015).

Obstructive apneas have been consistently shown to increase sympathetic outflow, assessed by means of plasma catecholamines, heart rate variability, and MSNA (Fatouleh et al., 2014). In obstructive sleep apnea periodicity is less evident and the HP usually shorter than in PB or Cheyne-Stokes respiration, also due to anatomical factors preventing the reopening of upper airways (Fatouleh et al., 2014; Butler et al., 2019; Hietakoste et al., 2020). On the other hand, the apnea length is more variable and may reflect individual differences in anatomical factors, chemoreflex sensitivity (low or high) or arousability (low or high) (Fatouleh et al., 2014; Butler et al., 2019; Hietakoste et al., 2020). While shorter apneas have been associated with increased mortality (Butler et al., 2019), longer ones have been associated with higher ultra-short term HRV (Hietakoste et al., 2020).

In patients with HFrEF and Cheyne-Stokes respiration apnea duration is usually less heterogenous, while HP length may vary, and this has been mainly attributed to differences in cardiac output and circulation time (Hall et al., 1996; Giannoni et al., 2019b; Gentile et al., 2022). Regarding the impact of CA on sympathetic outflow in the context of CSR the current evidence is contradictory (Lorenzi-Filho et al., 1999; Fatouleh et al., 2014; Oldenburg et al., 2015; Giannoni et al., 2019b). While some authors found CA to increase sympathetic outflow, others have attributed sympathetic outflow to depend solely on background hemodynamics and hence HFrEF severity (Burnum et al., 1954; Somers et al., 1989a; Maze et al., 1989; Hall et al., 1996; Solin et al., 2003; Oldenburg et al., 2007; Bitter et al., 2009; Wedewardt et al., 2010; Yumino et al., 2013; Cowie et al., 2015; Magalang and Pack, 2015; Cagnoni et al., 2016; Eulenburg et al., 2016; Emdin et al., 2017; Perger et al., 2017). Intermittent HP is an integral part of Hunter-Cheyne-Stokes respiration and may counterbalance the effects of CA on sympathetic outflow, possibly by ensuing hypocapnia-hyperoxia, but also by increasing cardiac output (Somers et al., 1989a; Somers et al., 1989b; Hall et al., 1996; Wedewardt et al., 2010; Seitz et al., 2013; Oldenburg et al., 2015). Thus, the overall impact of PB on sympathetic outflow, a major driver of HFrEF progression, may depend on the net effect of CA and HP and particularly by the relative duration of HP within the PB cycle.

This is of major clinical interest as two large multicentric studies using either continuous positive airway pressure or assisted servo-ventilation to treat CA in HFrEF showed neutral or even detrimental impact on survival (Bradley et al., 2005; Cowie et al., 2015; Eulenburg et al., 2016) In patients with HFrEF and Hunter-Cheyne-Stokes respiration apnea duration is usually less heterogenous, while HP length may vary, and this has been mainly attributed to differences in cardiac output and circulation time (Hall et al., 1996; Giannoni et al., 2019b; Gentile et al., 2022). Therefore, to investigate whether the duration of HP may specifically impact on sympathetic outflow, we instructed a group of healthy subjects to simulate two different oscillatory patterns of PB while measuring sympathetic outflow directly by means of intraneural MSNA recordings (Velez-Roa et al., 2004; Macefield, 2012; Macefield and Wallin, 2018; Macefield and Henderson, 2019) and indirectly via non-invasive assessment of heart rate variability (Oldenburg et al., 2015).

## 2 Methods

### 2.1 Study participants

Ten healthy participants (36 ± 9 [standard deviation] years, 5 male, 5 female, BMI 25 ± 2 kg/m^2^) were recruited. The study protocol conformed with the 1975 Declaration of Helsinki, other than registration in a database, and was approved by the institution’s Human Research Ethics Committee. All participants provided informed written consent to the studies, which were conducted in the Human Autonomic Neurophysiology laboratory at The Baker Heart and Diabetes Institute.

### 2.2 Study design

All participants underwent a thorough physical examination and evaluation of their past medical history to ensure the absence of previous diseases potentially affecting MSNA (Gratze et al., 1998; Maceira et al., 2006; Ponikowski et al., 2016).

### 2.3 Non-invasive haemodynamic and respiratory monitoring

Participants lay semi-recumbent in a chair with their back at 45^o^ and their legs supported horizontally. Electrocardiographic activity was recorded with Ag-AgCl surface electrodes (BioAmp, PowerLab, ADInstruments, Sydney, Australia) on the chest and sampled at 2 kHz (bandpass 0.3 Hz–1 kHz). Continuous blood pressure was recorded non-invasively by finger pulse plethysmography, sampled at 400 Hz (DC-200 Hz), and calibrated with an integrated sphygmomanometer cuff on the opposite upper arm (NOVA; Finapres Medical System BV, Amsterdam, Netherlands). Respiration was sampled at 100 Hz (DC-100 Hz) using a respiratory belt transducer (ADInstruments). All physiological signals were stored on computer via a data acquisition and analysis system (PowerLab 16SP™ hardware device and LabChart™ for Macintosh, v7.1.2.5 software; ADInstruments).

### 2.4 Assessment of sympathetic nerve activity

#### 2.4.1 Assessment of autonomic drive to the heart: Heart rate variability

For assessment of sympathovagal balance heart rate variability was analysed using a 3-lead electrocardiogram (sampling rate 200 Hz). HRV (expressed as ms^2^ based on continuously recorded variability in RR intervals) reflects sympathovagal balance and heart-brain interactions. Data were computed by frequency domain analysis and presented as the high frequency component (HF; 0.15–0.40 Hz), the low frequency component (LF; 0.04–0.15 Hz) and their relative ratio (LF/HF) (ESC Guidelines, 1996; Davies et al., 1999; Karemaker, 2017; Woehrle et al., 2017; Spiesshoefer et al., 2020; Spiesshoefer et al., 2021).

#### 2.4.2 Assessment of autonomic drive to the vasculature: Muscle sympathetic nerve activity

**FIGURE 1:**
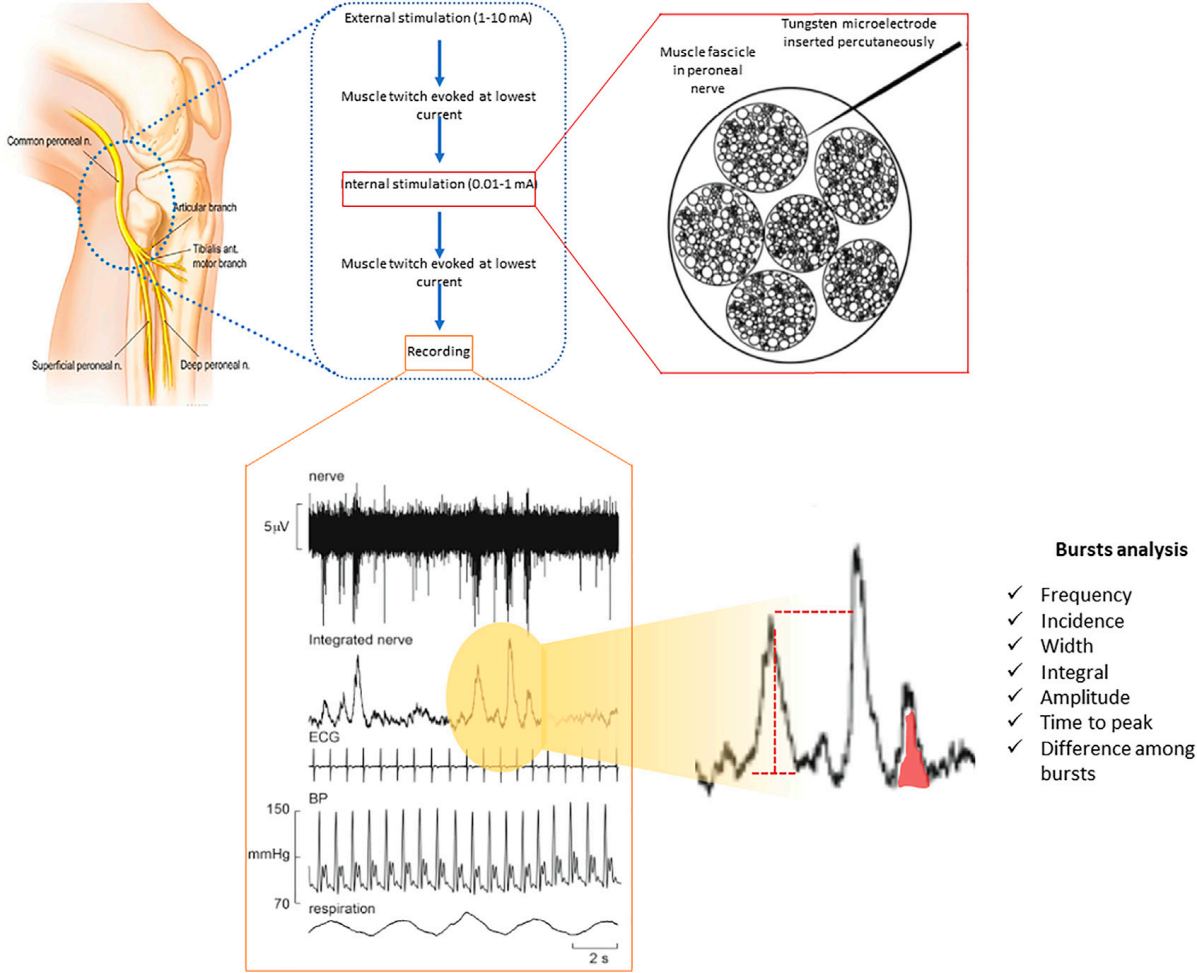
Multi-unit recording of muscle SNA from a healthy participant via a tungsten microelectrode inserted percutaneously into a muscle fascicle of the peroneal nerve, shown schematically on the left. Muscle SNA occurs as bursts with a clear cardiac rhythmicity. Heart rate and diastolic blood pressure variability can be derived from the setup used through ECG and continuous (finger) blood pressure all being recorded simultaneously.

Figure 1 displays the methodology to obtain and analyse MSNA measurements. Participants lay semi-recumbent in a chair with their back at 45^o^ and their legs supported horizontally. Electrical pulses (2–10 mA; Stimulus Isolator, ADInstruments, Sydney, Australia) were delivered via a 2 mm surface probe to locate the course of the right common peroneal nerve at the level of the fibular head. After identifying the optimal stimulation site, a sterile insulated tungsten microelectrode (Frederick Haer and Co., Bowdoin, United States) was inserted into the skin; an electrode with a larger uninsulated tip inserted at 1 cm distance served as the reference electrode. Weak electrical stimulation delivered through the active microelectrode (0.2 ms, 1 Hz, 0.01–1.0 mA) was used to guide manipulation of its tip into the nerve; evoked muscle twitches at 0.01–0.02 mA, without radiating paraesthesia, indicated that the tip had penetrated a muscle fascicle of the nerve.

Neural activity was amplified (gain 2 × 10^4^, bandpass 0.3–5.0 kHz) via an isolated amplifier and headstage (NeuroAmpEX, ADInstruments) and stored on computer (10 kHz sampling). Spontaneous or stretch-evoked activity of muscle spindle afferents, without afferent activity produced by light stroking of the leg or foot, confirmed the identity of the muscle fascicle of the nerve. The microelectrode was further manipulated until spontaneous bursts of muscle sympathetic nerve activity (MSNA) with clear cardiac rhythmicity, increasing during a maximal inspiratory apnoea, were observed.

A mean voltage neurogram of the filtered nerve signal was displayed from the root-mean square processed signal (200 ms moving average).

MSNA was analysed from the root mean square-processed nerve signal using the “Cyclic Measurements” and “Peak Parameters” features of the LabChart™ software: burst frequency (bursts per minute), burst incidence (bursts per 100 heart beats), burst width, burst area and time to peak (Figure 1) (Bradley et al., 2005; Macefield, 2012; Macefield and Henderson, 2019). In addition, amplitude distribution was determined: burst amplitudes were normalized to the highest burst amplitude seen during 1 minute of normal breathing (Bradley et al., 2005; Macefield, 2012; Macefield and Henderson, 2019).

### 2.5 Breathing maneuvers

After recording MSNA and cardiorespiratory signals during 10 min of normal breathing, participants were asked to follow different patterns of respiration displayed on a computer monitor in front of them. Each pattern was composed of sinusoidal waveforms of either constant amplitude and frequency (control) or two different types of PB as described below. These ventilatory patterns were generated in the LabChart™ software and displayed in the computer monitor so that participants could follow them in real time. Simulation of short and long HP cycle length were randomized in order. The patterns are shown in Figure 1. During the experiments participants’ own respiratory excursions were displayed below the emulated patterns. The different ventilatory manoeuvres were separated by 3 min of spontaneous breathing until MSNA, heart rate and blood pressure had returned to baseline.

#### 2.5.1 Simulation of periodic breathing

**FIGURE 2:**
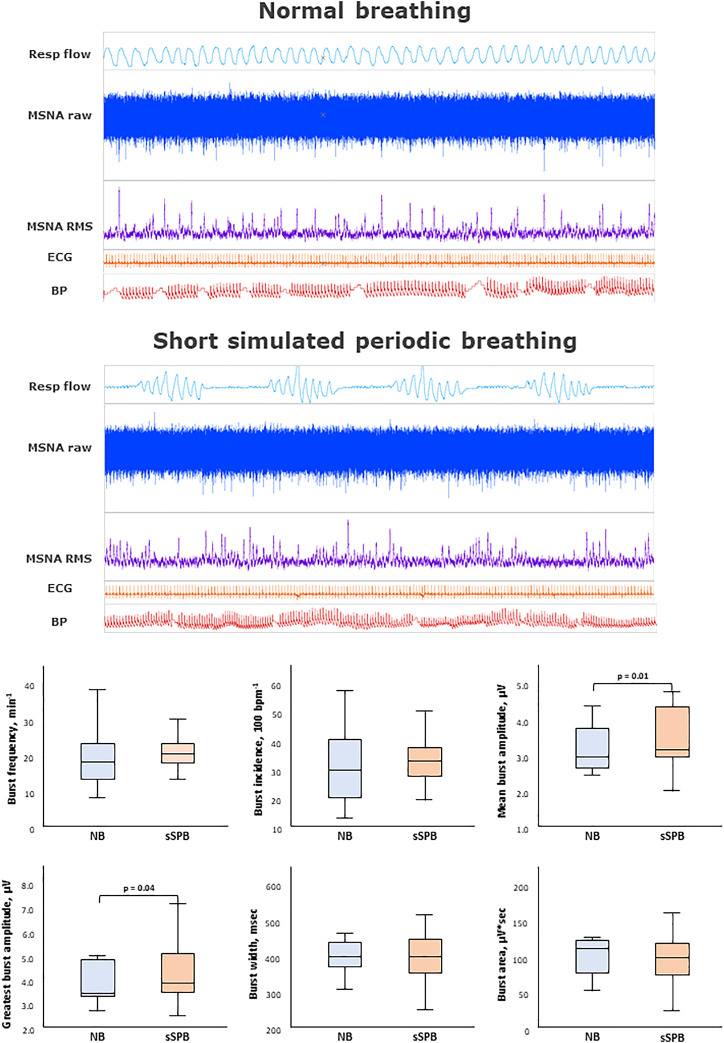
Impact of short simulated periodic breathing on muscle sympathetic nerve activity. Multi-unit recording of muscle SNA. RMS, root mean square, BP, blood pressure; Resp flow, respiratory flow.

Subjects were instructed to alternate HPs with CAs, thus simulating the typical crescendo-decrescendo pattern of PB. PB was simulated with 2 different HP lengths (20 and 40 s) while keeping the CA phase constant at 20 s duration, as in spontaneous PB in HFrEF, where PB cycle length depends on variable duration of the HP phase rather than apnea length (Solin et al., 2003; Magalang and Pack, 2015; Cagnoni et al., 2016; Perger et al., 2017). In addition, the simulated breathing patterns were specifically designed in such a way that apneas always started at end-expiration (referred to as a “negative PB breathing pattern” below), subsequently causing lung volume to fall below functional residual capacity during the hyperventilation phase. Participants were trained to practice several PB cycles by means of visual feedback before actual recordings were started (Figure 2).

### 2.6 Statistical analysis

Assuming a two-sided significance level of 0.05 (alpha) and 80% power (beta), a sample size of at least 10 patients was calculated for detection of a 10% change in MSNA burst rate and amplitude, respectively (mean values and standard deviations of MSNA burst rate and amplitudes for power calculations were derived from preliminary data). Secondary endpoints comprised the MSNA burst amplitude distribution (% amplitude as a ratio of the greatest MSNA burst seen in 1 min of normal breathing), burst width, integral, time to peak, time distance from the R wave and time distance among one another. Other secondary endpoints comprised systolic, diastolic and mean arterial blood pressure, heart rate and heart rate variability frequency domain metrics, and oxygen saturation.

All analyses were performed using Sigma Plot software (Version 13.0, Systat Software GmbH, Erkrath, Germany), PASS 14 Power Analysis and Sample Size Software 2015, respectively (NCSS, LLC. Kaysville, Utah, United States). Results were expressed as mean and standard deviation for continuous variables with a normal distribution, and median and interquartile range for continuous variables with a skewed distribution.

MSNA and cardiorespiratory parameters were compared using paired *t*-test or Wilcoxon Rank Sum Test, as appropriate. Before comparison, data were averaged across the 3-min intervals of the different PB (either short or long HP) manoeuvres, and the last 3 min of normal breathing that preceded the first respiratory task. When comparing the effects of CAs against HPs, data were again averaged including all phases of CAs, and all phases of HPs during the simulation task. Heart rate variability-derived metrics were also compared with MSNA-derived metrics employing Pearson correlation and Bland-Altman graphs. For all tests, a *p*-value ≤0.05 was considered statistically significant.

## 3 Results

**FIGURE 3:**
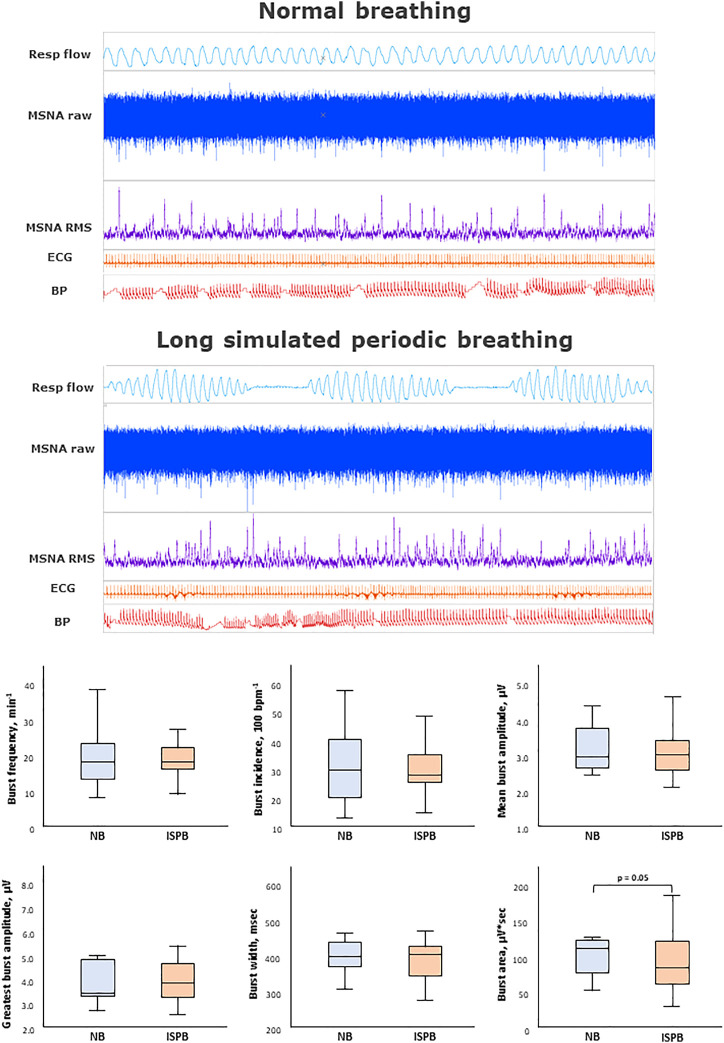
Impact of long simulated periodic breathing on muscle sympathetic nerve activity. Multi-unit recording of muscle SNA. RMS, root mean square, BP, blood pressure; Resp flow, respiratory flow.

**FIGURE 4:**
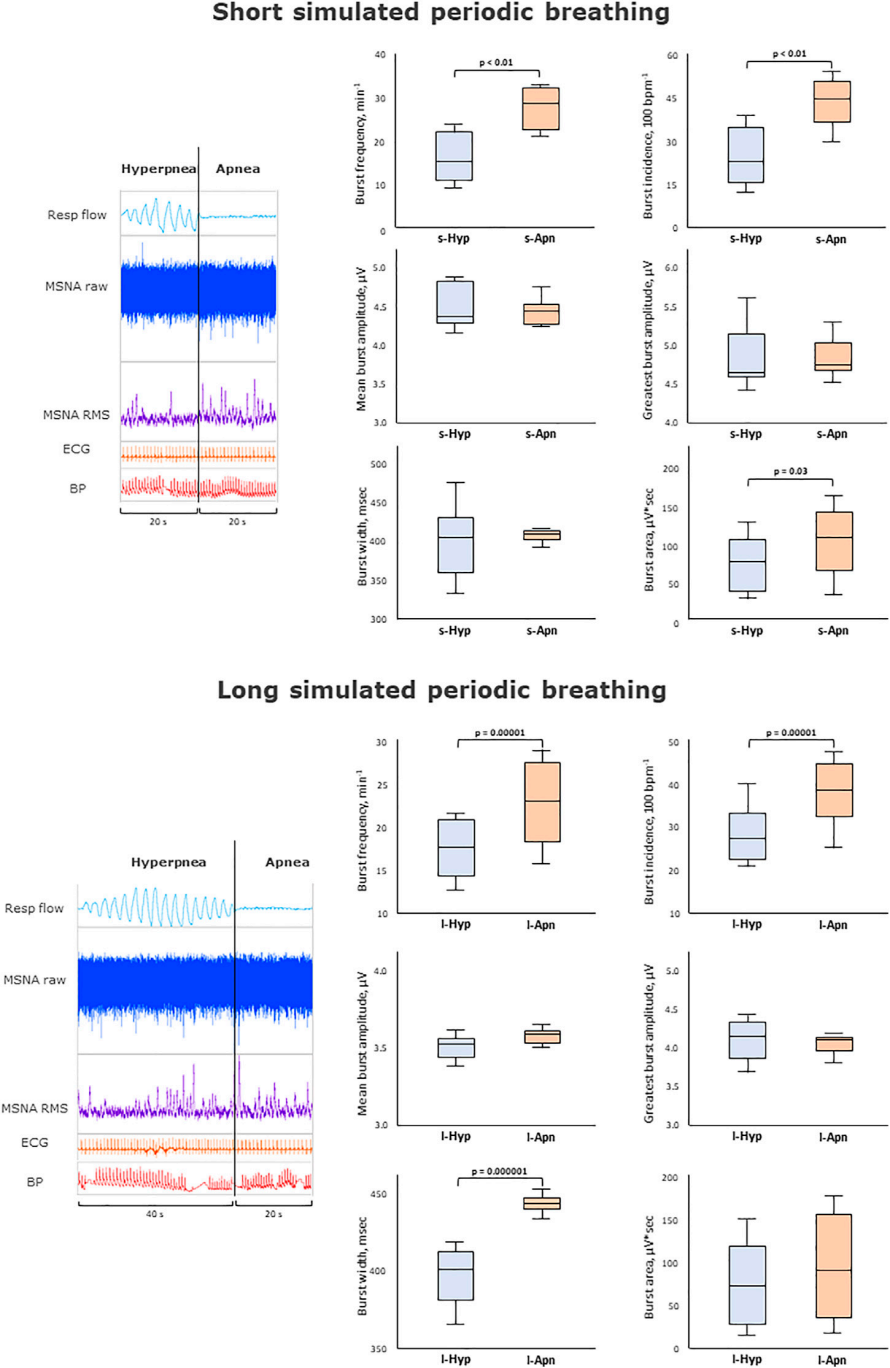
Impact of apneas compared to hyperpneas on muscle sympathetic nerve activity. Multi-unit recording of muscle SNA. RMS, root mean square, BP, blood pressure; Resp flow, respiratory flow.

The main results are shown in Figures 2–4 as graphical summaries.

### 3.1 Participants

All participants were normotensive, in sinus rhythm and without ectopic beats during all the recordings. No subject was taking any kind of medication possibly influencing sympathetic drive.

### 3.2 Baseline sympathetic outflow during normal breathing

In all participants and across all breathing manoeuvres a total of 1570 MSNA bursts were analysed. MSNA data, as well as heart rate variability-derived metrics, are shown in Table 1. Notably, during normal breathing, no correlations were found between spectral indices of heart rate variability and any of the MSNA-derived metrics.

**TABLE 1:** Comparison of sympathetic outflow, hemodynamic and respiratory data between normal breathing and periodic breathing with a short hyperpnea length.

	Baseline Normal breathing (average of 3 Min)	Periodic breathing with short HP (average of 3 Min)	*p*-value
Sympathetic Outflow parameters (MSNA)			
Burst frequency, min-1	19.2 ± 9.1	20.5 ± 4.8	0.32
Burst incidence, 100 bpm-1	32.0 ± 14.5	32.6 ± 8.1	0.78
Mean Burst Amplitude, μV	3.2 ± 0.8	3.4 ± 0.8	**0.02**
Greatest Burst Amplitude, μV	3.8 ± 0.9	4.3 ± 1.1	**0.01**
Burst Amplitude Distribution, %	84.2 ± 14.6	83.5 ± 14.5	0.84
Burst Width, msec	395.2 (368.2–437.6)	394.8 (327.9–445.0)	0.73
Burst Area, μV*msec	109.8 (76.2–122.0)	97.5 (72.2–118.3)	0.17
Burst time to Peak, msec	387.2 ± 180.5	345.8 ± 190.1	0.25
Burst difference among one another, sec	3.2 ± 1.0	3.1 ± 0.6	0.91
SD Burst difference among one another, sec	2.2 ± 0.9	2.6 ± 0.8	0.51
Sympathovagal balance parameters (HRV)			
HF RRI, ms^2^	647.8 ± 593.9	1081.9 ± 809.1	**0.01**
HFnuRRI, %	54 ± 22	44 ± 21	**0.02**
LF RRI, ms^2^	553.4 ± 570.5	1354.7 ± 1357.2	**0.0008**
LFnuRRI, %	46 ± 31	56 ± 41	**0.01**
LF/HF RRI	0.9 ± 0.5	1.7 ± 1.7	**0.03**
Haemodynamic parameters			
Heart rate, min-1	61.0 ± 5.8	63.7 ± 7.9	**0.0008**
Systolic BP, mmHg	126.0 ± 13.2	131.4 ± 25.4	0.30
Diastolic BP, mmHg	78.4 ± 26.3	61.7 ± 19.7	0.51
Respiratory parameters			
Respiratory rate, min-1	17.7 ± 2.3	30.5 ± 6.6	**0.0000001**
Oxygen saturation, %	98.9 ± 0.9	99.4 ± 0.7	**0.0004**

Values are mean ± standard deviation for data with a normal distribution, median (interquartile range) for data with a non-normal distribution, respectively. *p* values <0.05 displayed bold. HP, hyperpnea; MSNA; muscle sympathetic nerve activity; HRV; heart rate variability; SD, standard deviation; HF RRI, high frequency component of heart rate variability (nu; expressed as normalized units); LF RRI, low frequency component of heart rate variability (nu; expressed as normalized units); LF/HF RRI, relative ratio of low frequency and high frequency component of heart rate variability; BP, blood pressure.

### 3.3 Effects of short HP length on sympathetic outflow during simulated periodic breathing

Table 1 and Figure 2 provides an analysis of sympathetic outflow during simulation of PB with a short (20 s) HP length. An increase in both the mean and the highest burst amplitude was observed between the phases of normal breathing and simulated PB.

Other than this no differences were found in other MSNA parameters between normal breathing and simulated PB with short HP length, including burst frequency, incidence, width, integral, time to peak, or variability.

Despite the increase in respiratory rate and depth, there was no effect on blood pressure. On the contrary, an increase in heart rate, HF, LF, and LF/HF ratio was observed during PB with a short HP length. Likewise, respiratory rate, and oxygen saturation increased significantly during simulated PB (*p* < 0.01).

### 3.4 Effects of long HP length on sympathetic outflow during simulated periodic breathing

Table 2 and Figure 3 provides an analysis of sympathetic outflow response to simulation of PB with a long (40 s) HP length. Simulated PB with a long HP length decreased mean burst area as well as its standard deviation but otherwise had no effects on other MSNA or heart rate variability parameters. Heart rate and HF component of heart rate variability, respiratory rate and oxygen saturation increased significantly, while there was no change in blood pressure when comparing normal breathing to PB with long HP length.

**TABLE 2:** Comparison of sympathetic outflow, hemodynamic and respiratory data between normal breathing and periodic breathing with a long hyperpnea length.

	Baseline Normal breathing (average of 3 Min)	Periodic breathing with long HP (average of 3 Min)	*p*-value
Sympathetic Outflow parameters (MSNA)			
Burst frequency, min-1	19.2 ± 9.1	18.9 ± 4.3	0.81
Burst incidence, 100 bpm-1	32.0 ± 14.5	29.7 ± 7.5	0.36
Mean Burst Amplitude, μV	3.2 ± 0.8	3.1 ± 0.7	0.23
Greatest Burst Amplitude, μV	3.8 ± 0.9	3.9 ± 1.2	0.73
Burst Amplitude Distribution, %	84.2 ± 14.6	84.9 ± 9.9	0.69
Burst Width, msec	395.2 (368.2–437.6)	400.4 (339.0–423.9)	0.83
Burst Area, μV*msec	109.8 (76.2–122.0)	82.9 (55.5–122.6)	**0.05**
Burst time to Peak, msec	387.2 ± 180.5	375.5 ± 150.5	0.53
Burst difference among one another, sec	3.2 ± 1.0	3.2 ± 0.9	0.69
SD Burst difference among one another, sec	2.2 ± 0.9	2.5 ± 1.2	0.55
Sympathovagal balance parameters (HRV)			
HF RRI, ms^2^	647.8 ± 593.9	1605.1 ± 1746.7	**0.002**
HFnuRRI, %	54 ± 25	62 ± 30	**0.01**
LF RRI, ms^2^	553.4 ± 570.5	1016.9 ± 1265.1	0.08
LFnuRRI, %	46 ± 22	38 ± 21	0.07
LF/HF RRI	0.9 ± 0.5	1.2 ± 2.0	0.54
Haemodynamic parameters			
Heart rate, min-1	61.0 ± 5.8	64.3 ± 7.0	**0.00082**
Systolic BP, mmHg	126.0 ± 13.2	147.8 ± 35.8	0.46
Diastolic BP, mmHg	78.4 ± 26.3	84.2 ± 35.7	0.39
Respiratory parameters			
Respiratory rate, min-1	17.7 ± 2.3	25.9 ± 4.9	**0.000001**
Oxygen saturation, %	98.9 ± 0.9	99.5 ± 0.8	**0.0004**

Values are mean ± standard deviation for data with a normal distribution, median (interquartile range) for data with a non-normal distribution, respectively. *p* values ≤0.05 displayed bold. HP, hyperpnea; MSNA; muscle sympathetic nerve activity; HRV; heart rate variability; SD, standard deviation; HF RRI, high frequency component of heart rate variability (nu; expressed as normalized units); LF RRI, low frequency component of heart rate variability (nu; expressed as normalized units); LF/HF RRI, relative ratio of low frequency and high frequency component of heart rate variability; BP, blood pressure.

### 3.5 Effects of central apneas on sympathetic outflow during simulated periodic breathing

Table 3 and Figure 4 provides an analysis of MSNA changes between CAs and HPs during simulated PB. MSNA increased significantly during the apneic phase irrespective of HP length: bursts during apneas had a higher frequency and incidence and an increased burst area compared to the preceding hyperpneas.

**TABLE 3:** Comparison of sympathetic outflow and hemodynamic data between central apneas and hyperpnea phases during periodic breathing with a short and a long hyperpnea length.

	Normal breathing	Periodic breathing with short HP			Periodic breathing with long HP		
		Hyperpnoea	Apnoea	*p*-value	Hyperpnoea	Apnoea	*p*-value
Sympathetic Outflow (MSNA)							
Burst rate, min^−1^	19.2 ± 9.1	16.5 ± 5.6	27.5 ± 4.8	**>0.01**	17.6 ± 3.3	23.0 ± 4.6	**0.00001**
Burst incidence, 100 bpm^−1^	32.0 ± 14.5	25.9 ± 9.1	44.0 ± 6.9	**>0.01**	27.8 ± 5.4	38.4 ± 7.6	**0.00001**
Mean Burst Amplitude, μV	3.2 ± 0.8	4.5 ± 0.3	4.6 ± 0.3	0.60	3.6 ± 0.8	3.7 ± 0.4	0.14
Greatest Burst Amplitude, μV	3.8 ± 0.9	4.8 ± 0.5	4.78 ± 0.3	0.45	4.1 ± 0.3	4.0 ± 0.2	0.32
Burst Amplitude Distribution, %	84.2 ± 14.6	91.7 ± 9.2	90.4 ± 9.8	0.53	87.4 ± 8.3	88.8 ± 9.9	0.47
Burst Width, msec	395.2 (368.2–437.6)	402.5 ± 52.9 *	414.6 ± 22.9	0.15	399.8 ± 23.2 *	444.8 ± 10.8	**0.000001**
Burst Area, μV*msec	109.8 (76.2–122.0)	74.3 ± 45.8 *	104.8 ± 68.9	**0.03**	73.6 ± 45.2 *	95.6 ± 60.4	0.05
Burst time to Peak, msec	387.2 ± 180.5	282.6 ± 100.5	316.6 ± 100.8 *	0.13	291.0 ± 81.9 *	317.5 ± 109.2 *	0.06
Standard deviation of Burst difference among one another, sec	3.2 ± 1.0	5.4 ± 14.9	4.1 ± 13.9	0.67	10.2 ± 45.7	3.2 ± 5.8	0.49
Haemodynamic parameters							
Heart rate, min^−1^	—	63.4 ± 7.6	62.3 ± 7.6	0.39	66.5 ± 7.9 *	65.5 ± 8.9	0.12
Systolic BP, mmHg	—	151.7 ± 18.9	145.1 ± 20.2	0.16	162.2 ± 35.9	160.9 ± 35.1	0.58
Diastolic BP, mmHg	—	78.2 ± 14.9 *	73.4 ± 15.4 *	0.19	95.7 ± 34.1	95.7 ± 35.1	0.49

Values are mean ± standard deviation. *p* values (for paired *t* test hyperpneas vs central apneas) < 0.05 displayed bold. HPhyperpnea, HP, hyperpnea; MSNA; muscle sympathetic nerve activity; SD, standard deviation; BP, blood pressure. *Paired *t*-test or Wilcoxon for comparison versus baseline (normal breathing).

## 4 Discussion

This is the first study to determine the impact of simulated PB of different hyperpnea lengths as well as the immediate effects of CAs on sympathetic outflow in healthy subjects using MSNA, the gold standard technique for sympathetic outflow assessment. We demonstrated that end-expiratory CAs are associated with increased sympathetic outflow compared to HP, and that shorter PB cycles, in contrast to longer ones, are associated with an increase of sympathetic outflow, too. This seems to involve mainly the mean and the highest burst amplitude, at least in healthy individuals. Thus, the net effect of PB, measured over the entire respiratory cycle, derives from the equilibrium between the excitatory drive provided by CAs and the inhibitory effect provided by HPs: when the HP duration is short the relative balance of excitation vs. inhibition shift towards the first one.

This is of importance as PB is a highly prevalent phenomenon with a presumed incremental effect on sympathetic drive in HFrEF, as well as in conditions in the absence of HFrEF, with detrimental effects if left untreated (Burnum et al., 1954; Maze et al., 1989; Yumino et al., 2013; Magalang and Pack, 2015; Eulenburg et al., 2016; Perger et al., 2017). However, a previous study combining polysomnography and right heart catheterisation showed that the increase of sympathetic drive was related to the severity of HFrEF rather than to that of Cheyne-Stokes respiration (as reflected by the apnea-hypopnea index) (Mansfield et al., 2003a; Berry RB Gamaldo et al., 2012). Moreover, the unexpected results of the SERVE-HF trial suggested that treatment of CAs with adaptive servo-ventilation is associated with *increased* rather than decreased mortality in HFrEF patients with CAs, especially in those with severe HFrEF, namely with left ventricular ejection fraction lower than 30% (Cowie et al., 2015; Woehrle et al., 2017). This group of patients is likely to have also a decreased cardiac output and a longer circulatory time.

This has given rise to the hypothesis that CAs and PB potentially exert compensatory rather than detrimental effects in patients with HFrEF. Indeed, during hyperventilation, venous return and thus cardiac output are increased, and sympathetic drive is known to be inhibited by phasic (but not static) changes in lung volume and by hypocapnia (Burnum et al., 1954; Somers et al., 1989b; Hall et al., 1996; Wedewardt et al., 2010; Seitz et al., 2013; Oldenburg et al., 2015). Conversely, apneas are presumed to lead to increased sympathetic drive mainly in response to apnea-related hypoxemia and hypercapnia (Solin et al., 2003; Giannoni et al., 2016), but also to a lack of inhibitory drive from the stretch receptors located in the airways, lungs and chest wall.

In HFrEF, prolonged circulatory time *per se* prolongs both CA and HP duration (Macefield, 2012). Notably, patients with HFrEF and PB/Cheyne-Stokes respiration show longer HP and cycle length but similar apnea duration when compared with patients with HFrEF and obstructive sleep apnea (Macefield and Henderson, 2019), who usually have a less compromised heamodynamic profile (Hall et al., 1996; Wedewardt et al., 2010; Perger et al., 2017).

The present work suggests that in PB, HP duration may contribute to the net effect of sympathetic outflow.

Simulation of PB (in contrast to assessing its effects when it occurs spontaneously) has the advantage that the length and composition of PB cycles can be standardized, differently from the rather erratic and variable composition observed during spontaneous PB (central and obstructive apneas, mixed apneas, and variable cycle length). Thus, it is possible to precisely predefine the duration of the hyperventilation phase, while keeping CA constant in length. Second, the net effects of PB could be studied having phases of normal breathing as a reliable comparator. Our findings therefore, based on this approach, confirm that CA likely impacts negatively while HP likely impacts positively on sympathetic outflow. It is therefore, that a long cycle length of PB where CAs can be found embedded in long HP impacts neutral on sympathetic outflow, whereas this all results in increased sympathetic outflow should the same CAs be embedded into shorter HP. Furthermore, the present study also directly proved that CAs per se increase sympathetic outflow when compared directly against HP.

Indeed, CA may cause an increase in sympathetic outflow by several mechanisms: by deactivating pulmonary stretch receptors and stimulating peripheral and central chemoreceptors by hypoxia and hypercapnia (Floras, 2009). Furthermore, cortical activation following apneas may further rise central sympathetic outflow plus vagal withdrawal (Floras, 2009). The pathophysiological concept of CAs further increasing SNA is further supported by findings showing that patients with CAs are characterized by increased SNA as reflected by increased HR (Szollosi et al., 2007), decreased heart rate and blood pressure variability (Szollosi et al., 2007), enhanced serum and urinary catecholamine levels (Solin et al., 2003), as well as with increased MSNA (Solin et al., 2003) compared to patients without CAs. This theory is also further supported by studies linking the presence of CAs to worse prognosis in HFrEF with the presumed mechanism being increased SNA through CAs causing arrhythmias and HFrEF related death (Poletti et al., 2009). This has been shown for daytime CA and in HFrEF in particular (Emdin et al., 2017). Another exploratory approach to this key pathophysiological issue is to evaluate the effects of CA treatment on SNA. Indeed, reducing CA through administration of oxygen is also associated with SNA decrease (Staniforth et al., 1998). As for mask-based therapies based on positive pressure ventilation, despite their positive effect on CA in HF, there have been conflicting evidences on their impact on SNA too. While it was shown that cPAP and ASV lead to decreases in SNA as reflected by increased heart rate and blood pressure variability (Iwaya et al., 2014), decreased serum and urinary catecholamine levels (Hetzenecker et al., 2016; Toyama et al., 2017), as well as decreased MSNA (Koyama et al., 2010) other authors as well as a large scale RCT challenged these hypotheses (Cowie et al., 2015; Woehrle et al., 2017).

Nonetheless, the real impact of hyperpnea length may vary in patients with HFrEF, in whom several different pathophysiological mechanisms (chemoreflex gain, plant gain, circulation time, other sources of sympathetic outflow related to HFrEF itself or other altered circuits) may drive to different results. Therefore, an effort to study MSNA and other sympathetic outflow metrics should be attempted also in this relevant clinical setting before exporting our results to improve patients selection and referral to specific PB treatment. Ultimately, it should be noted that also conflicting evidence by other authors has been provided showing that CAs per se may not always result in increased sympathetic outflow (Mansfield et al., 2003b; Grimm et al., 2015).

### 4.1 Study limitations

Our study has several limitations. MSNA was obtained in 10 healthy participants with no cardiovascular or respiratory disease. Nevertheless, despite it being an invasive technique, one of the advantages of MSNA is undoubtedly its stability over time, requiring smaller sample size (Macefield, 2012; Macefield and Wallin, 2018; Macefield and Henderson, 2019) especially when studying the same participants in different experimental conditions.

Secondly, in our study PB was simulated. As noted above, PB cycle length is rather irregular in various pathologies and might therefore have a different impact on sympathetic outflow. Similarly, cortical influences secondary to simulation cannot be excluded. Studies focusing on spontaneous PB are therefore needed for more definite conclusions.

Third, the duration of each ventilatory manoeuvre was limited to 3 min. Therefore, we cannot exclude the possibility that longer periods of PB may exert different effects on the variables evaluated in this study, and that a drift in the average value of each haemodynamic measure may occur. Longer recordings are needed to clarify this point.

Fourth, considering that increasing the respiratory rate, the HF component may have been moved towards higher frequencies (>0.40 Hz underestimating HF component, by using the same frequency for everybody). Therefore a potential future study might use a wavelet analysis or a Wigner-Ville that may correct for non-stationarity of the signals (considering the respiratory maneuvers performed).

## 5 Conclusion

Apneas per se consistently resulted in markedly increased sympathetic outflow compared to phases of hyperventilation. However, at least in healthy subject simulating PB, the overall net effects of PB on on sympathetic outflow depend on hyperpnea length, with shorter duration of the hyperpneic phase resulting in increased sympathoexcitation. Future research should focus on the differential impact of longer recordings and focusing on patients with spontaneous PB during either wakefulness or sleep, considering at the same time the impact of several covariates including background haemodynamics, pulmonary mechanics, and the degree of feedback resetting.

## Data Availability

The original contributions presented in the study are included in the article/Supplementary Material, further inquiries can be directed to the corresponding author.
